# Supplementary material for: Nutrition and Rheumatoid Arthritis Onset: A Prospective Analysis Using the UK Biobank
Source: Nutrients. 2022 Apr 8;14(8):1554. doi: 10.3390/nu14081554 (PMC9025922; doi:10.3390/nu14081554)
Supplement: Supplementary file 1 [file nutrients-14-01554-s001.zip › nutrients-1613610-supplementary.pdf]

Supplementary Materials

Table S1. Distribution of missing values overall and by disease status.

Variable	Overall	RA	
		No	Yes
	N (%)	N (%)	N (%)
Sex	0 (0.00)	0 (0.00)	0 (0.00)
Age	0 (0.00)	0 (0.00)	0 (0.00)
Area	0 (0.00)	0 (0.00)	0 (0.00)
Townsend deprivation index	595 (0.12)	592 (0.12)	3 (0.11)
Smoking	1,957 (0.41)	1,942 (0.41)	15 (0.53)
BMI	2,498 (0.52)	2,466 (0.52)	32 (1.14)
Moderate/vigorous physical activity	10,364 (2.16)	10,237 (2.15)	127 (4.51)
Type II diabetes	0 (0.00)	0 (0.00)	0 (0.00)
Hypertension	0 (0.00)	0 (0.00)	0 (0.00)
Hypercholesterolemia	0 (0.00)	0 (0.00)	0 (0.00)
Other autoimmune diseases	0 (0.00)	0 (0.00)	0 (0.00)
Cooked vegetables	6,345 (1.32)	6,296 (1.32)	49 (1.74)
Salad/vegetables	6,583 (1.37)	6,530 (1.37)	53 (1.88)
Fresh fruit	2,227 (0.46)	2,209 (0.46)	18 (0.64)
Oily fish	3,004 (0.63)	2,978 (0.62)	26 (0.92)
Other fish	2,648 (0.55)	2,631 (0.55)	17 (0.60)
Processed meat	1,233 (0.26)	1,226 (0.26)	7 (0.25)
Chicken, turkey or other poultry	1,122 (0.23)	1,117 (0.23)	5 (0.18)
Beef	2,258 (0.47)	2,245 (0.47)	13 (0.46)
Lamb/mutton	3,388 (0.71)	3,358 (0.70)	30 (1.06)
Pork	3,187 (0.66)	3,160 (0.66)	27 (0.96)
Cheese	1,541 (0.32)	1,533 (0.32)	8 (0.28)
Bread	5,826 (1.22)	5,770 (1.21)	56 (1.99)
Breakfast cereals	1,955 (0.41)	1,944 (0.41)	11 (0.39)
Alcohol consumption	580 (0.12)	573 (0.12)	7 (0.25)
Tea	1,209 (0.25)	1,200 (0.25)	9 (0.32)
Coffee	1,263 (0.26)	1,248 (0.26)	15 (0.53)
Coffee type	107,358 (22.39)	106,619 (22.37)	739 (26.21)

RA: Rheumatoid Arthritis.
